# A case of anaphylactoid purpura nephritis accompanied by pulmonary hemorrhage and review of the literature

**DOI:** 10.3892/etm.2013.1020

**Published:** 2013-03-20

**Authors:** XIANQING REN, WENJUAN ZHANG, WEILI DANG, WENSHENG ZHAI, QINGYIN GUO, YIN DING, XIAOQING YANG

**Affiliations:** 1 Department of Pediatrics, The First Affiliated Hospital, Henan University of Traditional Chinese Medicine (TCM), Zhengzhou, Henan 450000, P.R. China; 2 Graduate School, Henan University of Traditional Chinese Medicine (TCM), Zhengzhou, Henan 450000, P.R. China

**Keywords:** anaphylactoid purpura nephritis, pulmonary hemorrhage, case report, adrenal cortex hormone, tripterygium glycosides

## Abstract

Cases of Henoch-Schönlein purpura and purpura nephritis accompanied by pulmonary hemorrhage are rare. Mild cases are easily ignored due to a lack of evident bleeding, and severe cases may be fatal. We have only treated one patient with Henoch-Schönlein nephritis (HSPN), a female child. The clinical manifestations were not evident, however, the imaging manifestations were clear. Finally, the patient was definitively diagnosed with HSPN accompanied by pulmonary hemorrhage. Following treatment with antiinflammatory and steroidal agents, tripterygium glycosides and traditional Chinese medicine, the patient recovered. In the present study, we report the diagnosis and treatment of this disease, with a review of the literature.

## Introduction

Henoch-Schönlein purpura (HSP) is a common vasculitis syndrome with systemic small-vessel vasculitis as the primary lesions. The main pathology is leukocytoclastic vasculitis of small dermal vessels and similar vasculitis characteristics are present in other sites, including the joints, gastrointestinal tract and kidneys (1). The main clinical manifestation of HSP is cutaneous purpura and this may be accompanied by abdominal pain and arthralgia. In addition, renal injury is also common. However, cases of HSP accompanied by pulmonary hemorrhage are rare. In China, there have been few studies on HSP and there have been scattered studies worldwide. In the clinic, HSP is usually ignored due to lack of evident bleeding. We have treated only one patient, a female child, with HSPN. The patient was finally diagnosed with HSP accompanied by pulmonary hemorrhage using a combination of laboratory examination, analysis of clinical manifestations and imaging examination. Following treatment, the disease condition was improved. In the present study we report on the diagnosis and treatment methods used for this disease, with a review of the literature. The study was conducted in accordance with the Declaration of Helsinki and with approval from the Ethics Committee of the First Affiliated Hospital, Henan University of Traditional Chinese Medicine (TCM; Zhengzhou, China). Written informed consent was obtained from the patient’s family.

## Case report

An 11-year-old female patient was hospitalized on April 3, 2012 due to cutaneous purpura of the lower limbs for half a month and urine test abnormality for three days. The patient presented with cutaneous purpura of the lower limbs without apparent cause (0.5–5 mm maximum diameter, bright red in color, skin swelling, no fading when compressed, and symmetric distribution). The patient also presented with knee and ankle joint pain and abdominal pain, but there were no symptoms of hematochezia, hematemesis or hemoptysis. Blood examination in The First Affiliated Hospital, Henan University of Traditional Chinese Medicine, showed that routine urine was normal. After the administration of hydrocortisone, cimetidine and amoxicillin for one week, the purpura disappeared and pain was relieved. The drug administration was immediately stopped while observations were conducted. Ten days following drug withdrawal, routine urine re-examination was conducted and gave the following results: urine protein (PRO) 3+; occult blood (BLD) 3+; and erythrocyte +++/HP. The patient was hospitalized on April 3, 2012 with HSPN at the First Affiliated Hospital, Henan University of Traditional Chinese Medicine (TCM). On admission, the lower limbs had no erythra or edema and there was no accompanying discomfort such as fever, abdominal pain, arthralgia, cough or expiratory dyspnea. Feces was normal and urine was deep yellow with appropriate volume.

Physical examination revealed the following: the body temperature was 36.5°C; the pulse was 96 bpm; the breathing frequency was 24 times/min; the blood pressure was 80/50 mmHg; and the body weight was 30 kg. The patient’s consciousness and mental state were normal. There was no erythra anywhere on the body and the pharyngeal cavity was hyperemic. Double lung auscultation indicated that the sound of the patient’s breath was clear, and dry and moist rales were not audible. Heart auscultation did not present any abnormality and the abdomen was soft and had no tenderness or rebound pain. The liver and spleen under the rib cage were palpable, the bowel sounds were normal and bilateral renal percussion was negative. The spine and joints of the four extremities did not present any deformity, and the four extremities did not present erythra or edema. The neurological examination revealed no abnormality.

A routine blood test revealed the following: a white blood cell (WBC) count of 5.1×10^9^ cells/l; a hemoglobin (HGB) level of 110 g/l; a red blood cell (RBC) count of 4.1×10^12^ cells/l; and a platelet (PLT) count of 260×10^9^/l (2012. 04. 04). The results of a routine urine test were as follows: PRO 3+, BLD 3+ and RBC 3+/HP. The fecal occult blood test was negative, the urine protein level at 24 h was 2.56 g/2000 ml urine and the urinary N-acetyl-β-D-glucosaminidase (NAG) enzyme level was 33.3 U/gCr. The coagulation functions were as follows: the prothrombin time was 10.5 sec, the international normalized ratio (INR, prothrombin time ratio) was 0.98, the fibrinogen level was 4.05 g/l, the activated partial prothrombin time was 36.1 sec (normal value, 24–36 sec) and the D-dimer level was 0.22 mg/l. The T-cell subset counts were: CD3^+^, 416/*μ*l; CD4+, 137/*μ*l; CD8^+^, 208/*μ*l; and CD4^+^/CD8^+^, 0.65/*μ*l. Liver and kidney function tests revealed the following: TP, 66.3 g/l; A, 38.7 g/l; alanine aminotransferase (ALT), 13.0 U/l; aspartate aminotransferase (AST), 24 U/l; blood urea nitrogen (BUN), 4.49 mmol/l; creatinine (Cr), 61.9 *μ*mol/l; and normal electrolyte levels. Humoral immunity, complement, C-reactive protein (CRP) and erythrocyte sedimentation rate (ESR) results were also normal; anti-nuclear antibody (ANA), extractable nuclear antibody (ENA), five indicators (HBsAg, HBsAb, HBeAg, HBeAb, HBcAb) of hepatitis B, three indicators (anti-HCV, anti-HIV, anti-TP) of infectious diseases and *Mycobacterium tuberculosis* antibody were negative. Double renal parenchyma presented mild diffuse changes, and the liver, gallbladder, spleen and pancreas showed no significant abnormality. Routine chest radiography indicated an irregular high-density shadow in the medium upper field of the right lung. Therefore, pulmonary hemorrhage was considered.

### Diagnosis and treatment process

**Figure 1:**
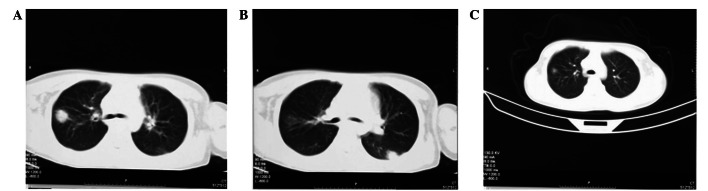
Pulmonary CT images prior to and following treatment. High density shadows were present in (A) the superior lobe dorsal segment of the right lung and (B) the inferior lobe of the left lung prior to treatment. (C) Following treatment, the original pulmonary hemorrhage was altered; it was clearly absorbed and reduced.

**Figure 2:**
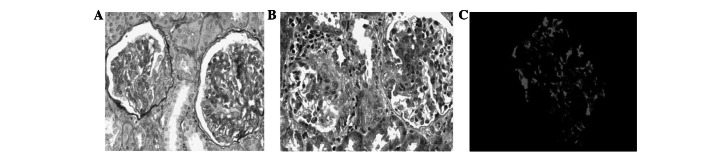
Pathological findings of renal biopsy. (A and B) under a light microscope (magnification ×400); (C) immunofluorescence. (B) Mesangial cell proliferation and crescent formation were visible, and (C) there were numerous IgA depositions on the mesentery and capillary walls.

Following admission, the patient’s disease history was examined. The patient had not recently presented with respiratory tract infections, including fever, cough, hemoptysis, pectoralgia, or hemorrhage. To further investigate the nature of the pulmonary high density shadow, a double lung CT examination was conducted. The result indicated that the inferior lobe of the left lung (Fig. 1B) and the superior lobe dorsal segment of the right lung presented high-density shadows (Fig. 1A), and the property was unclear, but the possibility of pulmonary hemorrhage was considered. Further examination results indicated that three serological tests of tuberculosis and the PPD test all were normal, and a T-spot assay indicated that interferon levels instituted by both antigen A and antigen B were zero, therefore, tuberculosis was not considered. Due to disease history, clinical manifestations, routine blood, blood sedimentation and CRP results, spherical pneumonia infection was also not considered. As the lesion nature remained undetermined, a lung aspiration biopsy was recommended, but the family refused. In addition, five indicators (PT, INR, APTT, TT, FIB) of coagulation were almost normal, and the 24-hour urine protein reached the criterion of massive proteinuria. Therefore, renal biopsy puncture was conducted. Renal pathological observation under a light microscope showed 12 glomeruli. Among them, there were two cellular crescents, and one glomerular segment that presented mesenteric and endothelial cell proliferation and lobulation. In addition, three glomerular segments presented mild mesenteric proliferation and focal segmental hypertrophy of podocytes, and two glomerular segments presented endothelial cell swelling. The basement membrane showed no significant abnormality, and the stroma presented a small amount of mononuclear cell infiltration (Fig. 2A and B). Immunofluorescence showed that the IgA (+++), C3 (+) mesentery and capillary vessels had small block- and particle-like deposits (Fig. 2C). Tests for IgG, IgM, HBsAg, HBeAg and HBcAg were negative, and the expression levels of type IV collagens α3 and α5 were normal. Therefore the patient was diagnosed with HSPN (IIIα) or pulmonary hemorrhage. The following treatment schemes were prepared: i) orally administered prednisone tablets, 2 mg/kg.day, three times daily; ii) tripterygium glycoside tablets, 2 mg/kg.day, three times daily; iii) Benazepril tablets, 10 mg/day; iv) due to the consideration of pulmonary hemorrhage, anticoagulant therapies, including heparin and Zantin were not used; v) traditional Chinese medicine was used for ‘clearing heat’ and ‘detoxifying and cooling blood’ and hemostasis. After 10 days of treatment, CT re-examination showed that the original pulmonary hemorrhage was altered; it was markedly absorbed and reduced (Fig. 1C). A routine urine test gave the following results: PRO 2+, BLD 3+, RBC 3+/HP and 24-hour urine protein, 0.99 g/day. The patient was recovering and so was discharged. The treatments were continued outside the hospital, and the doses of prednisone and tripterygium glycosides were gradually reduced. On August 19, 2012, the patient was visited and examined. The urine protein quantification at 24 h was 0.068 g; routine urine results were: PRO -, BLD 1+ and RBC 5–8/HP; and no recurrent purpura was visible.

## Discussion

HSP is a common clinical allergic disease in pediatric patients, and its main lesion is systemic small-vessel vasculitis. As numerous systemic small vessels are involved, multiple-system manifestations, including cutaneous purpura, joint swelling and pain, gastrointestinal symptoms and nephritis are visible. Among them, nephritis is the most important diagnostic indicator of HSP and it is the most common cause of mortality in patients with HSP. In addition, HSP has certain rare severe complications, including gastrointestinal bleeding, intussusception, intestinal perforation, intracranial hemorrhage and pulmonary hemorrhage. There are numerous reports of HSP accompanied by lung injury, but there are fewer reports of HSP accompanied by pulmonary hemorrhage. The literature reports that HSP accompanied by pulmonary hemorrhage is usually more severe in the clinic and easily causes mortalities. We definitively diagnosed an 11-year old female patient with anaphylactoid purpura nephritis accompanied by pulmonary hemorrhage. Further examination revealed a typical cutaneous purpura disease history, arthralgia, proteinuria and hematuria. A renal biopsy showed mesangial cell proliferation and crescent formation when viewed using light microscopy, and immunofluorescence indicated IgA deposition. X-ray signs of pulmonary hemorrhage were also present. However, as for clinical manifestations, there were no obvious cough, anhelation or expiratory dyspnea symptoms. Following the administration of oral prednisone tablets, tripterygium glycoside tablets, traditional Chinese medicine and general antiinflammatory treatment, the disease condition was relieved. The severity of the clinical manifestations in the present study is markedly different from that in the cases reported in the literature.

Lung injury is potentially present in all patients with HSP. The etiology of HSP is leukocytoclastic angiitis in the small vessels of the dermis. Therefore, HSP has numerous clinical symptoms, including pulmonary hemorrhage. The pulmonary hemorrhage is likely due to an allergic diffuse vasculitis, and may additionally be caused by an allergic reaction or immune function disorder. IgA immune complex deposition, fragmentation and the adhesion of a large number of white blood cells are the main causes of pulmonary hemorrhage, and they promote an increase in the permeability of the pulmonary capillary network to cause changes in pulmonary respiratory symptoms and chest X-rays. Kathuria and Cheifec (2) reported that microscopy showed HSP pulmonary alveolar hemorrhage, indicating leucocytolastic vasculitis with IgA deposition. However, in one 69-year-old patient reported by Usui *et al*(1), where the cause of mortality was HSP pulmonary hemorrhage, the lung tissue autopsy presented edema, extravasation of red blood cells and neutrophil infiltration of the alveolar wall, but there was no clear hemoleukocytic vasculitis manifestation, which was likely a result of steroid pulse therapy changing the pathology. These data indicate that the main pathogenic cause of the pulmonary hemorrhage in HSP is vasculitis and the hemorrhaging has little correlation with the coagulation function of patients. In the present study, the coagulation function indicators indicated a hypercoagulable state [prothrombin time, 10.5 sec; INR, 0.98; fibrinogen level, 4.05 g/l and D-dimer, 0.22 mg/l]. Due to the objections of family members, a pulmonary biopsy was not conducted. However, renal biopsy showed a large number of IgA deposits, confirming the previous analysis.

Although we are able to make a diagnosis according to HSP disease history, pulmonary typical manifestations and imaging symptoms, it remains difficult to make a differential diagnosis of HSP accompanied by pulmonary hemorrhage in the clinic since other vasculitis diseases, including systemic lupus erythematosus (SLE), Wegener’s granulomatosis and pulmonary hemorrhage-nephritis syndrome also have similar pulmonary hemorrhage manifestations. In addition, bacterial pneumonia, pneumonedema, pulmonary embolism and pulmonary tuberculosis also induce similar conditions, and are likely to be accompanied by HSP. Therefore, it is important to conduct immunological examinations, including antineutrophil cytoplasmic antibody (ANCN), ANA and ENA analyses. As lung biopsy has a certain risk for patients with this type of condition, it is necessary to identify important diagnostic information using methods including bronchoscopy, right cardiac catheterization and pulmonary arteriography. Certain authors suggest IgA deposition in the renal glomerulus to be the only reliable identification criterion for differentiating HSP accompanied by pulmonary hemorrhage from other vasculitis syndromes (3). Renal biopsy of the case reported in this study showed a large number of IgA deposits and the patient was finally diagnosed with anaphylactoid purpura nephritis accompanied by pulmonary hemorrhage. However, the authors consider that this indicator should be adopted more as a useful indicator, and that the use of multiple indicators is more appropriate for conducting a comprehensive judgment of the clinical diagnosis. In addition, it is necessary to identify clinical microscopic diagnosic indicators for HSP accompanied by pulmonary hemorrhage.

In a previous study, HSP accompanied by pulmonary hemorrhage has been divided into severe and mild types (4). Mild HSP accompanied by pulmonary hemorrhage presents mild pulmonary injury and/or renal injury, mostly with a single attack and favorable prognosis, and part of patients may naturally heal. However, severe cases rapidly present respiratory failure and renal inadequacy. Therefore, the mortality rate is high. In 15 cases of HSP accompanied by pulmonary hemorrhage reported in other studies, the patients all presented with severe multiple-organ injuries. Among them, six patients succumbed to pulmonary hemorrhage (5). In the present study, although the skin and joints manifested severe injuries, renal injury reached the pathological IIIα level and pulmonary imaging revealed a large shadow, the clinical respiratory manifestations were mild and no severe emergency events, such as bleeding or respiratory failure, occurred. It has been reported that HSP accompanied by pulmonary hemorrhage mainly occurs in young individuals and adults, and the mortality rate of adult patients with HSP accompanied by pulmonary hemorrhage is higher (5,6). In a study of 18 patients with HSP accompanied by pulmonary hemorrhage whose data were reported, three of 11 cases younger than 18 years old succumbed, and four of five cases older than 40 years old also succumbed (3), which indicated that the prognosis of the adolescent patients was better than that of the adult patients. In the present study, the patient was 11 years old and it was unclear whether her young age was the reason for milder clinical symptoms and a better prognosis. The existence of a correlation between age and prognosis and the cause for the correlation will be confirmed by further studies.

For the treatment of HSP accompanied by pulmonary hemorrhage, the majority of previous reports (1,5,8,9) suggest that the active use of steroids (oral prednisone tablets or methylprednisolone granules), immunosuppressants (cyclophosphamide, azathioprine and cyclosporine A) are able to markedly reduce the mortality rate. For 11 child patient cases reported in the literature, two out of six cases treated with methylprednisolone granules succumbed, three cases completely recovered and one case presented continuous proteinuria. The three cases treated with oral prednisone tablets and cyclophosphamide granules all presented continuous proteinuria and/or hematuria. One case treated with oral prednisone tablets combined with azathioprine completely recovered (3) and one case treated only with oral prednisone tablets succumbed. The above results indicate that the clinical efficacy of simple oral prednisone tablets is not ideal, and the effects of their combination with slow-action cyclophosphamide is also not ideal. In the present case, although acute inflammatory reactions, including abdominal pain and arthralgia, were more severe and renal injury reached medium severity at the beginning of onset, no cough, expiratory dyspnea, anemia and critical situations of failure of other organs were observed. Therefore, methylprednisolone granules were not used, and intravenous drip of cefatriaxone, oral prednisone and tripterygium glycoside tablets combined with Chinese herbal medicines were used for disintoxicating, promoting blood circulation and eliminating blood stasis. The efficacy of the combined treatments was good. In the treatment process of this disease case, in addition to prednisone, we also administered a mild immunosuppressant, tripterygium glycoside tablets was also used for treatment of this disease case. *Tripterygium wilfordii* is a traditional Chinese medicine. In 1997, it was first reported that its active components, tripterygium glycosides, had unique anti-inflammatory and immunosuppressive properties (10). Due to their high immunosuppressive efficacy and low incidence of side-effects, tripterygium glycosides are widely used for the treatment of autoimmune diseases and HSPN at present.

Cases of HSP accompanied by pulmonary hemorrhage are rare and HSP is typically ignored in the clinic due to a lack of evident bleeding. However, severe cases are acute and critical, and the mortality rate is high. Therefore, in clinical treatment of HSP, attention should be paid to pulmonary symptoms, in addition to common symptoms on skin, joint, urine and abdomen. Once unexplained expiratory dyspnea and hemoptysis appear in clinic, the possibility of accompanying pulmonary hemorrhage should be considered. When considering the treatment scheme of this disease, it is difficult to draw a definite conclusion as to whether the applied treatment scheme is suitable for other patients due to the lack of clinical cases. Therefore, further studies are required.
